# Cocktail of REGN Antibodies Binds More Strongly to SARS-CoV-2 Than Its Components, but the Omicron Variant Reduces Its Neutralizing Ability

**DOI:** 10.1021/acs.jpcb.2c00708

**Published:** 2022-04-11

**Authors:** Hung Nguyen, Pham Dang Lan, Daniel A. Nissley, Edward P. O’Brien, Mai Suan Li

**Affiliations:** † Institute of Physics, Polish Academy of Sciences, al. Lotnikow 32/46, 02-668 Warsaw, Poland; ‡ Life Science Lab, Institute for Computational Science and Technology, Quang Trung Software City, Tan Chanh Hiep Ward, District 12, 729110 Ho Chi Minh City, Vietnam; § Faculty of Physics and Engineering Physics, VNUHCM-University of Science, 227, Nguyen Van Cu Street, District 5, 749000 Ho Chi Minh City, Vietnam; ∥ Department of Statistics, University of Oxford, Oxford Protein Bioinformatics Group, OX1 2JD Oxford, United Kingdom; ⊥ Department of Chemistry, Penn State University, University Park, Pennsylvania 16802, United States; # Bioinformatics and Genomics Graduate Program, The Huck Institutes of the Life Sciences, Penn State University, University Park, Pennsylvania 16802, United States; ∇ Institute for Computational and Data Sciences, Penn State University, University Park, Pennsylvania 16802, United States

## Abstract

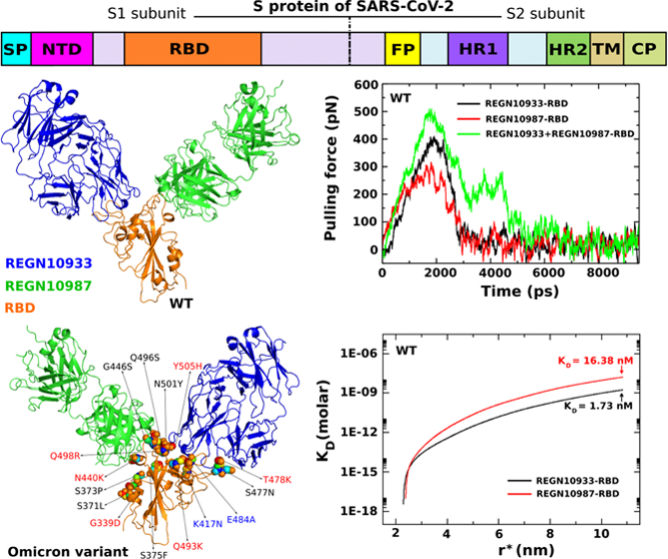

A promising approach to combat Covid-19 infections is the development of effective antiviral antibodies that target the SARS-CoV-2 spike protein. Understanding the structures and molecular mechanisms underlying the binding of antibodies to SARS-CoV-2 can contribute to quickly achieving this goal. Recently, a cocktail of REGN10987 and REGN10933 antibodies was shown to be an excellent candidate for the treatment of Covid-19. Here, using all-atom steered molecular dynamics and coarse-grained umbrella sampling, we examine the interactions of the receptor-binding domain (RBD) of the SARS-CoV-2 spike protein with REGN10987 and REGN10933 separately as well as together. Both computational methods show that REGN10933 binds to RBD more strongly than REGN10987. Importantly, the cocktail binds to RBD (simultaneous binding) more strongly than its components. The dissociation constants of REGN10987-RBD and REGN10933-RBD complexes calculated from the coarse-grained simulations are in good agreement with the experimental data. Thus, REGN10933 is probably a better candidate for treating Covid-19 than REGN10987, although the cocktail appears to neutralize the virus more efficiently than REGN10933 or REGN10987 alone. The association of REGN10987 with RBD is driven by van der Waals interactions, while electrostatic interactions dominate in the case of REGN10933 and the cocktail. We also studied the effectiveness of these antibodies on the two most dangerous variants Delta and Omicron. Consistent with recent experimental reports, our results confirmed that the Omicron variant reduces the neutralizing activity of REGN10933, REGN10987, and REGN10933+REGN10987 with the K417N, N440K, L484A, and Q498R mutations playing a decisive role, while the Delta variant slightly changes their activity.

## 1 Introduction

Severe acute respiratory syndrome coronavirus 2 (SARS-CoV-2), is a member of the Coronaviridae family and the causative agent of the ongoing coronavirus disease 2019 (Covid-19) pandemic.^1^ Currently, over 245 million cases have been officially diagnosed since its first emergence, and more than 5 million people have died from Covid-19.^2^ Public health measures, along with rapid vaccine development, have helped slow the pandemic in some countries. Moreover, small-molecule inhibitors, antibody-based therapeutics, and convalescent plasma from recovered Covid-19 patients have received emergency use approvals.^3^

**Figure 1:**
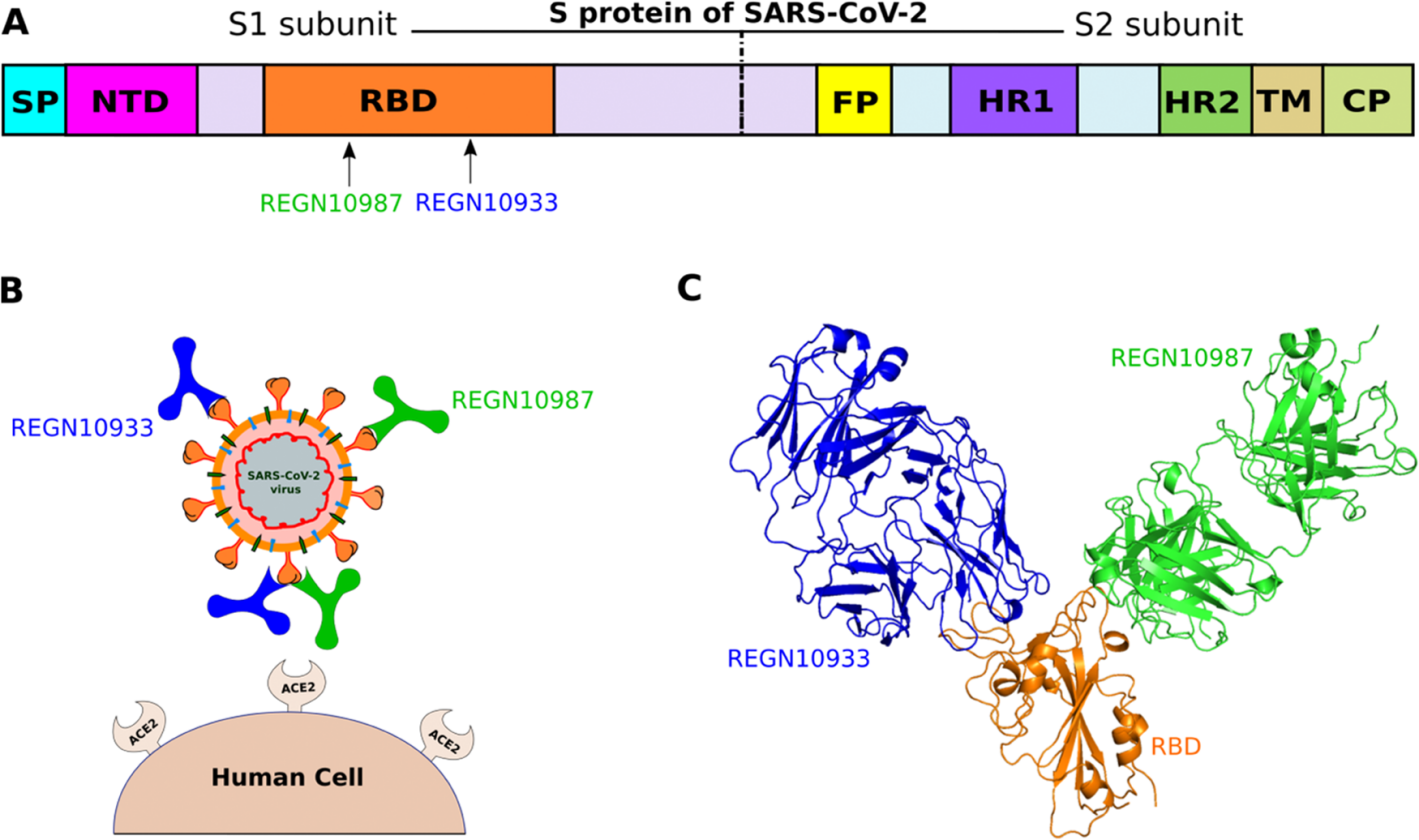
(A) Schematic description of the S protein of SARS-CoV-2, which consists of S1 and S2 subunits. (B) REGN10933 and REGN10987 bind to S protein, preventing the virus from entering cells. (C) Three-dimensional (3D) structures of REGN10933 and REGN10987 bound to RBD are shown in all-atom representation.

Monoclonal antibody (mAb) therapies for the treatment of SARS-CoV-2 have proven to be an excellent solution to reduce virus loads and alleviate symptoms when given shortly after diagnosis.^4,5^ mAbs bind to the virus through the spike protein (S), which consists of the S1 and S2 subunits (Figure 1A), blocking the binding of SARS-CoV-2 to human angiotensin-converting enzyme 2 (ACE2) (Figure 1B), in turn preventing infection.^6^ mAbs often target the S1 subunit,^7^ which contains the receptor-binding domain (RBD) and N-terminal domain (NTD). RBD-specific mAbs fall into four main classes, while NTD-specific mAbs target the patch remote from RBD.^8,9^ The discovery of mAbs that target S2 is another area of active research.^8^

Antibody cocktails, defined as mixtures of more than one unique antibody, have shown promise in preventing viruses from escaping neutralization *in vitro*.^10,11^ Recently, a double antibody cocktail (REGN-COV2) for SARS-CoV-2, including REGN10933 and REGN10987, has entered phase 2/3 clinical trials. This can be seen as the REGN-COV2 therapy developed by Regeneron Pharmaceuticals in which both monoclonal antibodies bind to RBD (Figure 1C).^12,13^ REGN10933 tethers at the top of RBD, significantly overlapping the binding site of ACE2 (dissociation constant *K*_D_ = 3.37 nM), while REGN10987 is located lateral to RBD, away from the REGN10933 epitope and has little to no overlap with the ACE2 binding site (*K*_D_ = 45.2 nM) (Table 1).^12^*In vitro* studies showed that combining two noncompeting antibodies protects against the rapid escape seen with individual antibody components.^13^ This combination-based approach has been supported by subsequent studies showing that REGN-COV2 retains neutralization potency against SARS-CoV-2.^12,13^

**Table 1:** *K*_D_ (nM) of REGN-COV2 Antibodies Bound to RBD for the WT Case Estimated from the Experimental and Computational Results

WT			
		*K*_D_ (simulation)	
	*K*_D_ (experiment)	REX-US	PRODIGY
REGN10933	3.37	1.73	31 ± 8.96
REGN10987	45.2	16.38	69 ± 25.33
REGN10933+REGN10987			0.056 ± 0.027

Despite reports of the important role of REGN-COV2 in the treatment of Covid-19, the structure and mechanism of binding of REGN-COV2 antibodies to RBD at the atomic level have not been studied. In this work, we use steered molecular dynamics (SMD) and coarse-grained simulations with umbrella sampling to evaluate the binding affinities of REGN10933, REGN10987 and both REGN10933+REGN10987 to RBD. Our theoretical estimation of the dissociation constant agrees with the experiment, according to which *K*_D_ of REGN10933-RBD is less than that of REGN10987-RBD (Table 1). Both SMD and PRODIGY (PROtein binDIng enerGY prediction) show that REGN10933+REGN10987 binds to RBD more tightly than its components.

More recently, many experimental studies on SARS-CoV-2 variants such as α, β, γ, Delta, Lambda, Omicron, etc. have shown that these variants can promote the ability to infect host cells and evade host immunity, which means that they will increase binding to ACE2 and weaken the neutralizing capacity of most SARS-CoV-2 antibodies.^14−24^ However, there are some antibodies that recognize and bind to the S protein of these variants, blocking the virus from infecting human cells. For example, a cocktail of antibodies REGN10933 and REGN10987 can neutralize most variants of SARS-CoV-2 including α, γ, Delta, and so on.^25,26^ The Omicron variant reported in November 2021 could reduce the effectiveness of a monoclonal antibody cocktail in treating Covid-19.^20,21^ Therefore, understanding the molecular mechanism underlying the activity of SARS-CoV-2 variants is essential to find an appropriate and timely therapy for Covid-19.

Since the Delta and Omicron variants play a major role in viral infection, we investigated their interaction with REGN-COV2. We found that the binding affinities of REGN10933, REGN10987, and REGN10933+REGN10987 to the Delta variant remain almost the same as those of the wild type (WT). However, the Omicron variant significantly decreases the interaction with REGN-COV2, which is consistent with the experiment.^20,21^ Our comprehensive study provides important mechanistic insights into the stability of the respective complexes, which can be useful for the development of antibody cocktail therapy for Covid-19.

## 2 Materials and Methods

### 2.1 Preparing the Structures

The structure of the REGN-COV2 antibody cocktail with two components REGN10933 and REGN10987 bound to RBD (Figure 1C) was obtained from the Protein Data Bank, PDB ID: 6XDG.^12^ Missing residues were added using the Modeler package.^27^ In this work, we considered the Delta (B.1.617.2) and Omicron (B.1.1.529) variants. All mutations of these variants were generated using the mutagenesis tool in PyMOL package.^28^

### 2.2 All-Atom Simulation

Simulations were carried out with the CHARMM36 force field^29^ in the GROMACS 2016 package^30^ at a temperature of 310 K and a pressure of 1 bar, which were maintained using the v-rescale and Parrinello–Rahman algorithms.^31,32^ The TIP3P water model^33^ was used to solvate all structures. All bonds within proteins were constrained by the Linear Constraint Solver (LINCS) algorithm.^34^ Electrostatic and van der Waals interactions were used to depict nonbonded interactions and their pair list is updated every 10 fs with a cutoff of 1.4 nm. The Particle Mesh Ewald algorithm^35^ was used to calculate the long-range electrostatic interaction. The equations of motion were solved using the leap-frog algorithm^36^ with an integration time step set to 2 fs. Periodic boundary conditions were applied in all directions. The energy of these systems was minimized using the steepest-descent algorithm and then equilibrated with a short 2 ns simulation performed in the NVT ensemble, followed by 3 ns NPT simulation. Finally, a 100 ns production simulation was performed to generate initial conformations for SMD simulation and for the estimation of the binding free energy using structure-based PRODIGY. Five statistically independent trajectories were run for each system.

#### 2.2.1 Steered Molecular Dynamics

**Figure 2:**
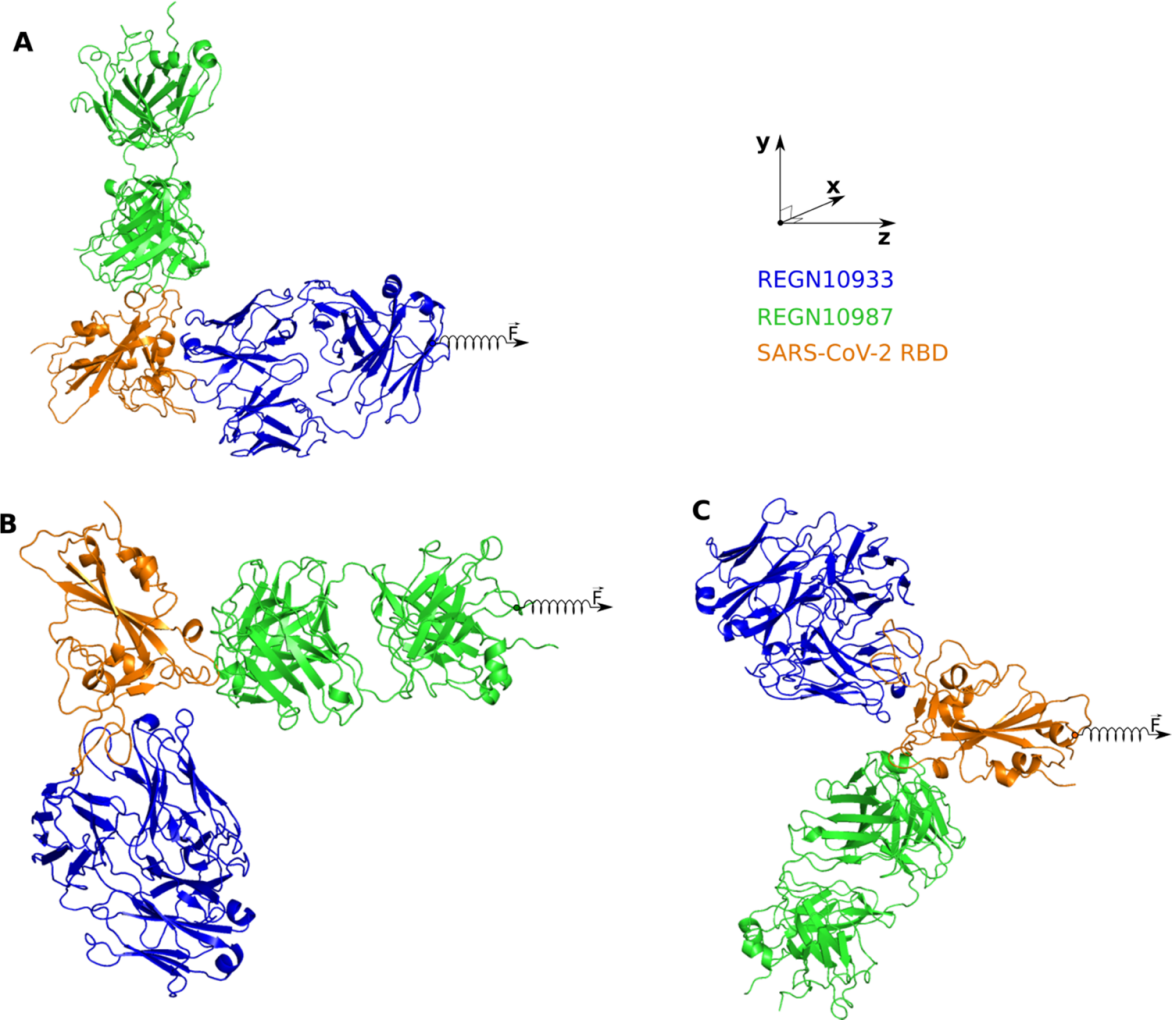
Structure of the REGN10933+REGN10987-RBD complex, retrieved from PDB with ID 6XDG. RBD is shown in orange, while green and blue describe REGN10987 and REGN10933. The external force is applied to (A) REGN10933, (B) REGN10987, and (C) RBD (REGN10933+REGN10987). The pulling direction in SMD simulations is shown with a spring along the *z*-axis.

A rectangular box of 10 × 16 × 25 nm^3^ was used to allow enough space to pull the targets from their binding regions. The center of three-dimensional coordinates was at 5 × 8 × 6 nm^3^ for these complexes. K^+^ and Cl^–^ ions were added to a concentration of 0.15 M. In the case of REGN10933-RBD and REGN10987-RBD, an external force is applied to a dummy atom, which is linked to the Cα atom closest to the antibody center of mass (CoM). The pulling direction is parallel to the vector connecting CoMs of the RBD and antibody (Figure 2A,B). In the case of REGN10933+REGN10987-RBD, the pulling direction is the line connecting RBD’s CoM in perpendicular to the line connecting the CoMs of REGN10933 and REGN10987 (Figure 2C). These complexes were then rotated so that the REGN10933-RBD or REGN10987-RBD or REGN10933+REGN10987-RBD unbinding pathway is along the *z*-axis (Figure 2), which was displayed using the PyMOL 2.0 package.^28^ The force experienced by the pulled atom is measured according to the following equation
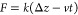
1where *k* is the force constant, *v* is the pulling velocity at time *t*, and Δ*z* is the displacement of the chain’s atom connected to the spring in the direction of pulling, respectively. The spring constant *k* value was set to 600 kJ/(mol nm^2^) (∼1020 pN/nm), which is a typical value used in atomic force microscopy (AFM) experiments.^37^ The complete dissociation of REGN10933 or REGN10987 or RBD from the binding region was reached during simulations of duration ∼10,000 ps at pulling speed *v* = 0.5 nm/ns.

Using the force–displacement profile gained in the SMD simulation, nonequilibrium work (*W*) was estimated using the trapezoidal rule
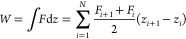
2where *N* is the number of simulation steps, and *F_i_* and *z_i_* are the force experienced by the target and position at step *i*, respectively. To estimate the binding free energy (Δ*G*) from the SMD simulation, we used Jarzynski’s equality^38^ in the presence of external force with constant pulling speed *v*. The Δ*G* was defined by^39^
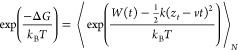
3here, ⟨...⟩_*N*_ is the average over *N* trajectories, *z*_*t*_ is the time-dependent displacement, and *W*(*t*) is the nonequilibrium work at time *t* defined as eq 2.

Equation 3 means that we can extract an equilibrium quantity by assembling the external work of an infinite number of nonequilibrium processes.^40^ In this study, while the transformation is not slow enough and the number of SMD runs is finite, we are able to estimate the nonequilibrium binding and unbinding energy barriers of the complexes based on the transition state (TS), the bound state (at *t*_0_), and the unbound state (at *t*_end_).

#### 2.2.2 Measures Used in Data Analysis

A hydrogen bond (HB) defined by the distance between donor D and acceptor A is less than 0.35 nm, the H–A distance is less than 0.27 nm, and the D–H–A angle is larger than 135°. A nonbonded contact (NBC) between two residues of a protein was made considered to be present when the distance between their heavy atoms is 0.39 nm or less. The two-dimensional (2D) contact networks of HBs and NBCs of REGN10933-RBD and REGN10987-RBD were analyzed using the LIGPLOT package.^41^

### 2.3 Coarse-Grained Simulations

#### 2.3.1 Coarse-Grained Model for Proteins

Protein was described using the Go-like model. Each amino acid is represented by a single interaction site positioning at the corresponding C_α_ coordinates. The configuration energy is calculated as below
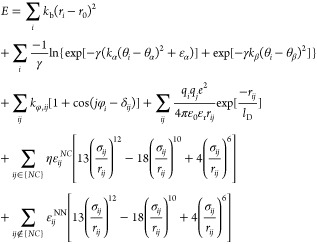
4

These terms represent, respectively, the energy contributions of C_α_–C_α_ bonds, bond angles, dihedral angles, electrostatics, and Lennard-Jones (LJ)-like attractive and repulsive interactions of native and nonnative contacts. Details of parameters employed for these terms can be found elsewhere.^42^ Lennard-Jones (LJ) well depths for native contact interactions were set by a scaling factor η to reproduce realistic protein stabilities. η values for intraprotein interactions of antibodies and RBD domain were defined through the procedure described previously based on a published training set.^43^ An additional η is set for the inter-interactions between the antibody and RBD to reproduce the dissociation constant *K*_D_ at the nanomolar level reported by experiments. η values are listed in Table S1.

#### 2.3.2 Replica Exchange Umbrella Sampling (REX-US) Simulations

Here, we employed Chemistry at Harvard Macromolecular Mechanics (CHARMM) version c35b5 to perform Replica Exchange Umbrella Sampling (REX-US) coarse-grained simulations to explore the binding of two antibodies REGN10933 and REGN10987 to RBD. In total, 200 umbrella windows were generated by translating the antibody in 0.05 nm increments away from RBD along the vector connecting their two interface centers of mass. A harmonic restraint with a force constant of 700 kcal/(mol nm^2^) was applied to restrain the antibody and virus domain at target distances. Langevin dynamics simulations were then run at 310 K using a frictional coefficient of 0.050 ps^–1^, an integration time step of 0.015 ps, and the SHAKE algorithm^44^ applied to virtual bonds. Exchanges between neighboring windows were attempted every 5000 integration time steps (75 ps). In total, 10,000 exchanges (750 ns of simulation time) were run with the acceptance ratios between neighboring umbrellas falling in the range of 0.46–0.79. The first 1000 attempted exchanges were discarded to allow for equilibration, and the remaining 9000 exchanges used for analysis.

#### 2.3.3 Method for Estimating the Dissociation Constant (*K*_D_) from REX-US Simulations

The dissociation constant *K*_D_ is calculated as below^45,46^
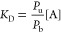
5where [A] represents the concentration of the free antibody or free RBD in their unbound state. *P*_u_ is the probability of the system being in the unbound state, *P*_u_ = 1–*P*_b_. The bound-state probability *P*_b_ is calculated from the numerical integration of the one-dimensional potential of mean force (1D-PMF) *G*_1D_ (*r*) as below
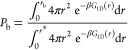
6

where *G*_1D_ (*r*) was constructed from REX simulations using WHAM equations.^47^

### 2.4 Structure-Based Method to Predict the Binding Affinity of Antibodies

MD-based exact methods, such as free energy perturbation or thermodynamics integration, can provide highly accurate results, but due to high computational costs, their application is restricted to study the small compound binding or effect of mutations, which requires high precision. Docking methods based on the knowledge of the three-dimensional (3D) structure of associated molecular complexes are more commonly used due to their wide range of applicability, although the accuracy depends on structural characteristics. The result is obtained mainly from the contribution of surface interactions. Recently, more research has been conducted to improve the structure-based prediction for the protein–protein binding affinity. Taking into account the contribution of characteristics of the noninteracting surface, Vangone and Bovine^48^ described the binding affinity of two interacting proteins by an analytically linear equation. The combination of polar–nonpolar charge residues is sorted by the contribution of interresidue contacts. The buried surface area and the noninteracting surface effect are computed separately for polar–nonpolar residues. The corresponding weights are obtained by training different combinations of proteins whose binding affinities have been experimentally measured. This method is currently implemented as a web server tool PRODIGY (PROtein binDIng enerGY prediction) with a software version deposited on GitHub repository.^49^ Here, we used PRODIGY to predict the binding affinity of the systems under study for comparison with the results obtained from our coarse-grained simulations and experiments.

## 3 Results and Discussion

### 3.1 Hydrogen Bonded and Nonbonded Contact Networks

We analyzed the HB and NBC networks of REGN10933-RBD and REGN10987-RBD of the initial structure to gain some insight into the binding affinity between REGN-COV2 antibodies and RBD (Figure S1). There were 15 and 8 residues of REGN10933 and REGN10987, respectively, that have formed HB or NBC contacts with RBD. While seven HBs are formed between REGN10933 and RBD, no HBs are formed between REGN10987 and RBD. A total of 15 NCBs formed between REGN10933 and RBD as well as between REGN10987 and RBD. The difference in the number of HBs suggests that the binding affinity of REGN10933 to RBD may be stronger than that of REGN10987 to RBD.

### 3.2 Steered Molecular Dynamics Simulation Results

#### 3.2.1 Ranking of Binding Affinities of REGN-COV2 Antibodies to RBD: REGN10987 < REGN10933 < REGN10933+REGN10987

**Figure 3:**
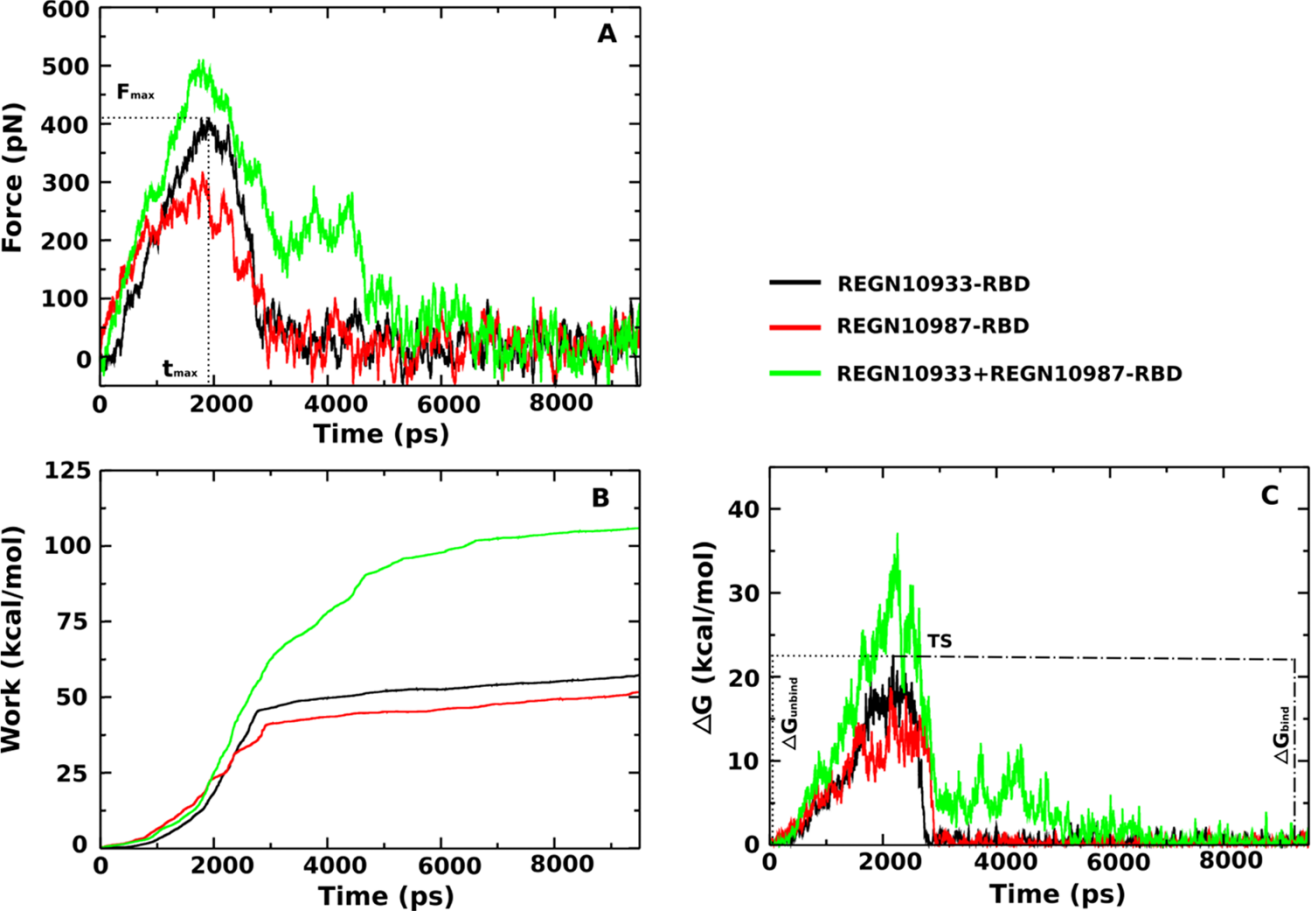
Time dependence of (A) force, (B) pulling work, and (C) nonequilibrium free energy of the REGN10933-RBD, REGN10987-RBD, and REGN10933+REGN10987-RBD complexes. The results were averaged over five independent SMD runs.

The force, pulling work, and free energy barrier profiles of REGN10933-RBD, REGN10987-RBD, and REGN10933+REGN10987-RBD are shown in Figure 3. Averaging over five independent runs, for REGN10933-RBD and REGN10987-RBD, we obtained *F*_max_ ≈ 411.0 and 318.3 pN, respectively (Figure 3A and Table 2), which are lower than that of REGN10933+REGN10987-RBD (511.3 pN).

**Table 2:** Rupture Force (*F*_max_), Time Rupture (*t*_max_), Nonequilibrium Work (*W*), and Nonequilibrium Binding Energy Barriers (ΔΔ*G*_bind_ and ΔΔ*G*_unbind_) Obtained from Five Independent SMD Trajectories of REGN10933-RBD, REGN10987-RBD, and REGN10933+REGN10987-RBD for the WT and the Variants^a^

	REGN10933-RBD			REGN10987-RBD		REGN10933+REGN10987-RBD		
	WT	Delta	Omicron	WT	Omicron	WT	Delta	Omicron
*F*_max_ (pN)	411.0 ± 21.3	424.0 ± 25.6	326.2 ± 21.1	318.3 ± 19.5	279.3 ± 14.3	511.3 ± 20.2	529.2 ± 22.5	436.4 ± 21.7
*t*_max_ (ps)	1915.3 ± 117.0	2007.5 ± 120.3	1557.0 ± 109.8	1800.9 ± 111.2	1684.3 ± 104.8	1739.6 ± 108.5	1638.2 ± 101.6	2426.7 ± 126.7
*W* (kcal/mol)	57.3 ± 1.5	56.9 ± 1.8	45.2 ± 1.7	51.6 ± 1.4	41.8 ± 1.0	105.8 ± 2.7	111.2 ± 2.2	65.5 ± 1.9
ΔΔ*G*_unbind_ (kcal/mol)	22.7 ± 1.7	22.0 ± 1.1	12.8 ± 1.0	18.7 ± 0.5	11.2 ± 0.2	37.1 ± 1.5	37.2 ± 1.3	30.1 ± 0.4
ΔΔ*G*_bind_ (kcal/mol)	22.1 ± 1.3	21.6 ± 1.4	12.7 ± 1.4	18.4 ± 0.7	11.1 ± 0.3	37.0 ± 1.7	37.1 ± 1.5	30.0 ± 0.9

a Here, the errors represent standard deviations.

The nonequilibrium work *W* increased until the antibodies detached from RBD and then saturated. Therefore, *W* is defined as the saturated value at the end of the simulation. In detail, *W* = 57.3 ± 1.5, 51.6 ± 1.4, and 105.8 ± 2.7 kcal/mol for REGN10933-RBD, REGN10987-RBD, and REGN10933+REGN10987-RBD, respectively (Figure 3B and Table 2).

The nonequilibrium binding free energy (Δ*G*) for three complexes is estimated from eq 3. Clearly, we have Δ*G*_bound_ = Δ*G*(*t*_0_) ≈ 0 kcal/mol at the beginning of the bound state, while the unbound state occurs at the end of the simulation, Δ*G*_unbound_ = Δ*G*(*t*_end_) ≈ 0 kcal/mol. The binding and unbinding free energy barriers are defined by ΔΔ*G*_bind_ = Δ*G*_TS_ – Δ*G*_unbound_ and ΔΔ*G*_unbind_ = Δ*G*_TS_ – Δ*G*_bound_, where Δ*G*_TS_ is the maximum free energy corresponding to the transition state. Then, from Figure 3C, we have ΔΔ*G*_unbind_ = 22.7 ± 1.7, 18.7 ± 0.5, and 37.1 ± 1.5 kcal/mol and ΔΔ*G*_bind_ = 22.1 ± 1.3, 18.4 ± 0.7, and 37.0 ± 1.7 kcal/mol for REGN10933-RBD, REGN10987-RBD, and REGN10933+REGN10987-RBD, respectively (see also Table 2).

Thus, the data obtained for *F*_max_, *W*, ΔΔ*G*_bind_, and ΔΔ*G*_unbind_ (Table 2) indicate that REGN10933 binds to RBD more strongly than REGN10987. Moreover, the REGN10933+REGN10987 cocktail associates with the spike protein more closely than the individual components, resulting in a ranking of REGN10987 < REGN10933 < REGN10933+REGN10987. It can be expected that after binding to the S protein, two antibodies will physically occupy the ACE2 interaction interface (see Figure S2) and completely block the ACE2-S interaction, which will lead to the fact that the virus neutralization process will be faster than the neutralization process of one of them separately.

#### 3.2.2 Stabilities of REGN10933-RBD and REGN10933+REGN10987-RBD are Driven by Electrostatic Interactions, While the Stability of REGN10987-RBD is Controlled by vdW Interaction

**Figure 4:**
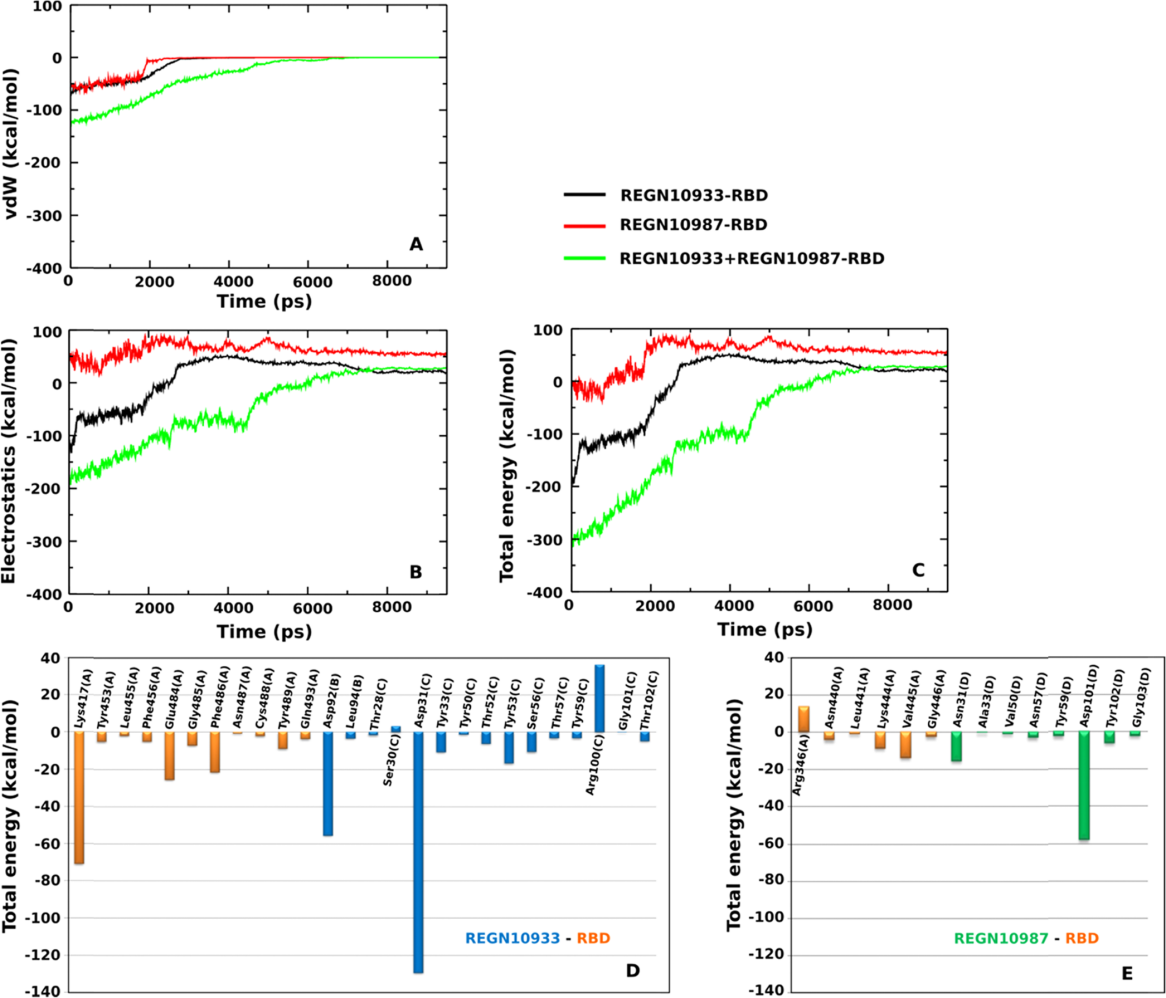
Time dependence of (A) vdW interaction energy, (B) electrostatic interaction energy, and (C) total interaction energy (sum of electrostatic and vdW interactions) of the REGN10933-RBD, REGN10987-RBD, and REGN10933+REGN10987-RBD complexes. (D, E) Total interaction energies of the residues at the binding regions of REGN10933 and REGN10987 with RBD (Figure S1). Results were obtained in the time window [0, *t*_max_] and averaged over five independent SMD runs.

The time dependence of the energy of electrostatic (*E*_elec_), van der Waals (*E*_vdW_), and total (*E*_total_, the sum of electrostatic and vdW) interactions is illustrated in Figure 4A–C. The results are averaged over five SMD trajectories. In the bound state, *E*_elec_ of REGN10933-RBD and REGN10933+REGN10987-RBD started with a negative value, while for REGN10987-RBD, it fluctuated at a positive value. However, for the last complex, *E*_vdW_ was negative (Figure 4A), resulting in *E*_total_ < 0 (Figure 4C). In the unbound state, due to the long-range character, *E*_elec_ reached a positive value for all three systems. On the other hand, their *E*_vdW_ was negative in the bound state, and then eventually reached 0 in the unbound state.

Since the complex has been in the bound state before the rupture occurs, the interaction energy of this state can be obtained by averaging over the time window [0, *t*_max_]. This gives us *E*_elec_ = −65.7 ± 4.3, 44.2 ± 3.4, and −155.9 ± 4.4 kcal/mol, *E*_vdW_ = −51.1 ± 1.8, −50.8 ± 2.4, and −107.2 ± 3.2 kcal/mol for REGN10933-RBD, REGN10987-RBD, and REGN10933+REGN10987-RBD, respectively (Table 3). Then, *E*_total_ = −116.8 ± 6.1, −6.6 ± 5.8, and −263.1 ± 7.6 kcal/mol for REGN10933-RBD, REGN10987-RBD, and REGN10933+REGN10987-RBD, respectively. It is obvious that REGN10987-RBD is marginally stable in terms of the interaction energy without regard to entropy and is less stable than the other two complexes. In addition, the electrostatic interaction makes an important contribution to REGN10933-RBD and REGN10933+REGN10987-RBD, while the vdW interaction plays a key role in REGN10987-RBD binding.

**Table 3:** Nonbonded Interaction Energies (kcal/mol) of REGN10933-RBD, REGN10987-RBD, and REGN10933+REGN10987-RBD Complexes for the WT^a^

	REGN10933-RBD	REGN10987-RBD	REGN10933+REGN10987-RBD
*E*_elec_	–65.7 ± 4.3	44.2 ± 3.4	–155.9 ± 4.4
*E*_vdW_	–51.1 ± 1.8	–50.8 ± 2.4	–107.2 ± 3.2
*E*_total_	–116.8 ± 6.1	–6.6 ± 5.8	–263.1 ± 7.6

a The results were obtained for a [0–*t*_max_] time window and averaged from five SMD trajectories.

#### 3.2.3 Role of Specific Residues in the Binding Regions of REGN10933-RBD and REGN109387-RBD

To calculate the per-residue interaction energy in the bound state, we took into account the images collected in the window [0, *t*_max_] and averaged over all SMD trajectories. The results obtained for the residues from the REGN10933-RBD and REGN10987-RBD binding regions are shown in Figure 4D,E.

Assuming that important residues must have an interaction energy, the absolute value of which exceeds 20 kcal/mol, then for REGN10933-RBD residues Asp92(B), Asp31(C), and Arg100(C) of REGN10933 and Lys417(A), Glu484(A), and Phe486(A) of RBD make a major contribution. The letters in the brackets refer to the chains. With an interaction energy of about −71.1 kcal/mol, Lys417(A) of the spike protein is much more significant than Glu484(A) (−25.7 kcal/mol) and Phe486(A) (−21.6 kcal/mol) (Figure 4D). Negatively charged residues Asp92(B) and Asp31(C) from REGN10933 stabilize the complex, while positively charged Arg100(C) destabilize it with a positive energy.

In the REGN10987-RBD case, the interaction energy is much lower compared to the REGN10933-RBD complex, and only the Asp101(D) residue of REGN10987 has an energy below −20 kcal/mol (Figure 4E). However, the greatest influence on REGN10933 binding is exerted by Arg346(A), Lys444(a), and Val445(A) of RBD.

Since the total charge of SARS-CoV-2-RBD is +2e, the negatively charged residues of both REGN10933 (Asp92(B) and Asp31(C)) and REGN10987 (Asp101(D)) substantially increase their binding affinity with RBD. This means that an antibody that contains many negatively charged residues at the interface with the spike protein is powerful in blocking a viral infection.

#### 3.2.4 Delta Variant has a Little Effect on the Binding Affinities of REGN10933, REGN10987, and REGN10933+REGN10987 with RBD

Recent studies have indicated that mutated residues in RBD directly affected the neutralizing activity of most antibodies against Covid-19 variants.^15−26,50^ In this work, we conducted SMD simulations for the most dangerous Delta and Omicron variants (Table S2) to shed light on the molecular mechanisms underlying the influence of mutated residues on the neutralizing ability of the REGN-COV2 cocktail.

**Figure 5:**
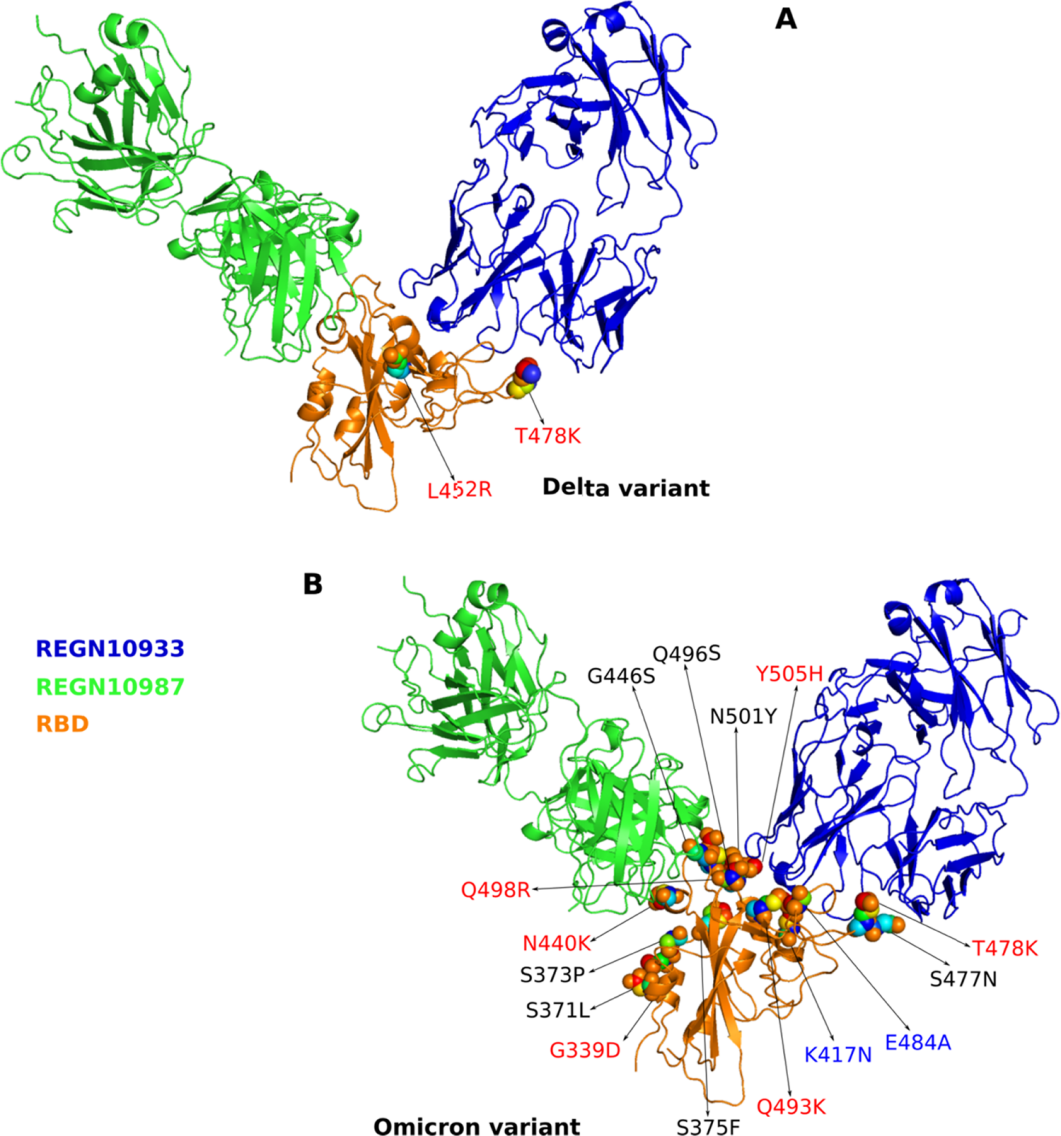
Mutations of (A) Delta and (B) Omicron variants in RBD. The mutations of the Omicron variant in RBD are at the binding regions for both constituents of the REGN-COV2 cocktail, while the mutations of the Delta variant in RBD influence only the REGN10933 binding site. Blue refers to residues that have charge in RBD-WT, while red denotes residues that have charge after mutation.

**Figure 6:**
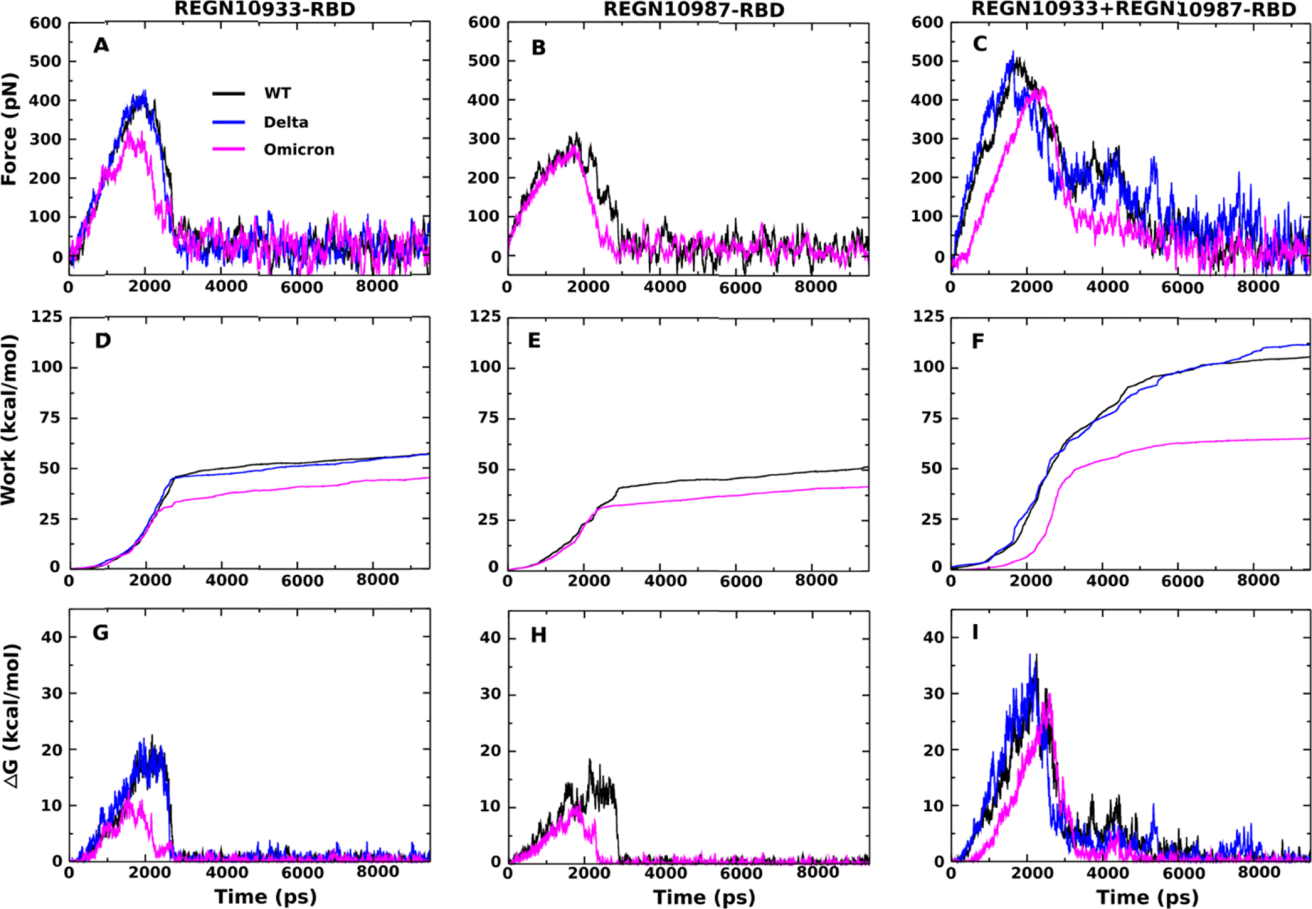
Time dependence of (A–C) pulling force, (D–F) pulling work, and (G–I) nonequilibrium free energy of the complexes. These results averaged over five independent SMD runs for WT and the variants.

As can be seen from Figure 5A, only REGN10933 has contact with RDB at L452 and T478 residues, where the mutation was made for the Delta variant. Therefore, SMD simulation was carried out for REGN10933-RBD and REGN10933+REGN10987-RBD, but not for REGN10987-RBD. Upon mutation, the RBD charge increases from +2e to +4e (Table S3), but these mutations have a little effect on the interaction between antibodies and the spike protein. In particular, *F*_max_, *W*, Δ*G*_bind_, and Δ*G*_unbind_ of the Delta variant are close to those of WT (Figure 6 and Table 2), implying that, as for WT, REGN10933 and REGN10933+REGN10987 are also effective for this variant. This is because the L452R and T478K mutations do not significantly contribute to the REGN10933-RBD stability, as their total interaction energy varies from −0.5 and −3.4 kcal/mol (WT) to 0.4 and −3.5 kcal/mol (Delta) (Table 4).

**Table 4:** Total Interaction Energies (kcal/mol) of the Important Residues of RBD to REGN10933 and REGN10987 for the WT and the Variants^a^

mutation points on RBD		
WT	Delta	Omicron
G339: −0.1		D339: −2.5
S371: 0.03		L371: 0.1
S373: −0.2		P373: −0.1
S375: −0.03		F375: −0.04
K417: −71.1		N417: −5.0
N440: 0.1		K440: 6.4
G446: 0.02		S446: −0.8
L452: −0.5	R452: 0.4	
S477: −9.9		N477: −8.8
T478: −3.4	K478: −3.5	K478: −5.5
E484: −25.7		A484: −1.9
Q493: −3.9		K493: −8.5
G496: −0.3		S496: −2.1
Q498: 1.6		R498: 16.7
N501: −2.1		Y501: −1.2
Y505: −0.02		H505: 1.3

a The results were obtained in a [0, *t*_max_] time window averaged from five SMD trajectories.

As can be seen from Table 2 (columns 7 and 8), when two mAbs are combined, they show no difference in the binding affinity between the reference strain and the Delta strain, but they are known not to offer the same level of protection clinically. To clarify this issue from a biophysical point of view, we docked ACE2 to the complex of REGN10933, REGN10987, and RBD using the HDOCK software (Figure S2).^51,52^ Since the distance between the centers of mass of RBD and ACE2 for the WT case (2.37 nm) is greater than for Delta (2.19 nm), two antibodies can prevent ACE2 from binding to the WT RBD to a greater extent than to the Delta RBD, which can lead to different protection activities. This effect is understandable if we take into account the ACE2-RBD attractive electrostatic interaction, which is stronger for Delta than for WT because ACE2 has a charge of −26e, while the charges of Delta RBD and WT RDB are +4e and +2e, respectively (Figure S2).

#### 3.2.5 Omicron Variant Attenuates the Binding Affinities of REGN10933, REGN10987, and REGN10933+REGN10987 with RBD

For the Omicron variant, all 15 mutated residues in RBD interact with both REGN10933 and REGN10987 (Figure 5B). Therefore, we performed SMD simulations for all three complexes REGN10933-RBD, REGN10987-RBD, and REGN10933+REGN10987-RBD. After mutation, the RBD charge increases from +2e (WT) to +5e (Omicron) (Table S3), which enhances the repulsive interaction with positively charged REGN10933 (+3e) and REGN10987 (+6e) (Table S3), resulting in reduced binding affinity of the Omicron variant. This prediction has been confirmed by the SMD results obtained for three complexes (Figure 6 and Table 2). *F*_max_, *W*, Δ*G*_bind_, and Δ*G*_unbind_ of Omicron are clearly lower than those of WT, suggesting that REGN10933, REGN10987, and REGN10933+REGN10987 are less effective against this variant. To better understand this problem, we calculated the interaction energy of each mutated residue in RBD. The decrease in the interactions of REGN10933 and REGN10987 with RBD is mainly due to the K417N, N440K, E484A, and Q498R mutations, which increases the interaction energy at these positions from −71.1, 0.1, −25.7, and 1.6 kcal/mol (WT) up to – 5.0, 6.4, −1.9, and 16.7 kcal/mol (Omicron) (Table 4). Although the total interaction energy of Q493K decreased from −3.9 kcal/mol (WT) to −8.5 kcal/mol (Omicron), this contribution is not enough to change the overall behavior of REGN-COV2 toward RBD in the Omicron variant. Thus, among the 15 mutations, K417N, N440K, E484A, and Q498R play a key role in reducing the effectiveness of REGN-COV2 antibodies against the Omicron variant.

### 3.3 Coarse-Grained Simulation Results

#### 3.3.1 REGN10933 Binds to RBD More Strongly than REGN10987

**Figure 7:**
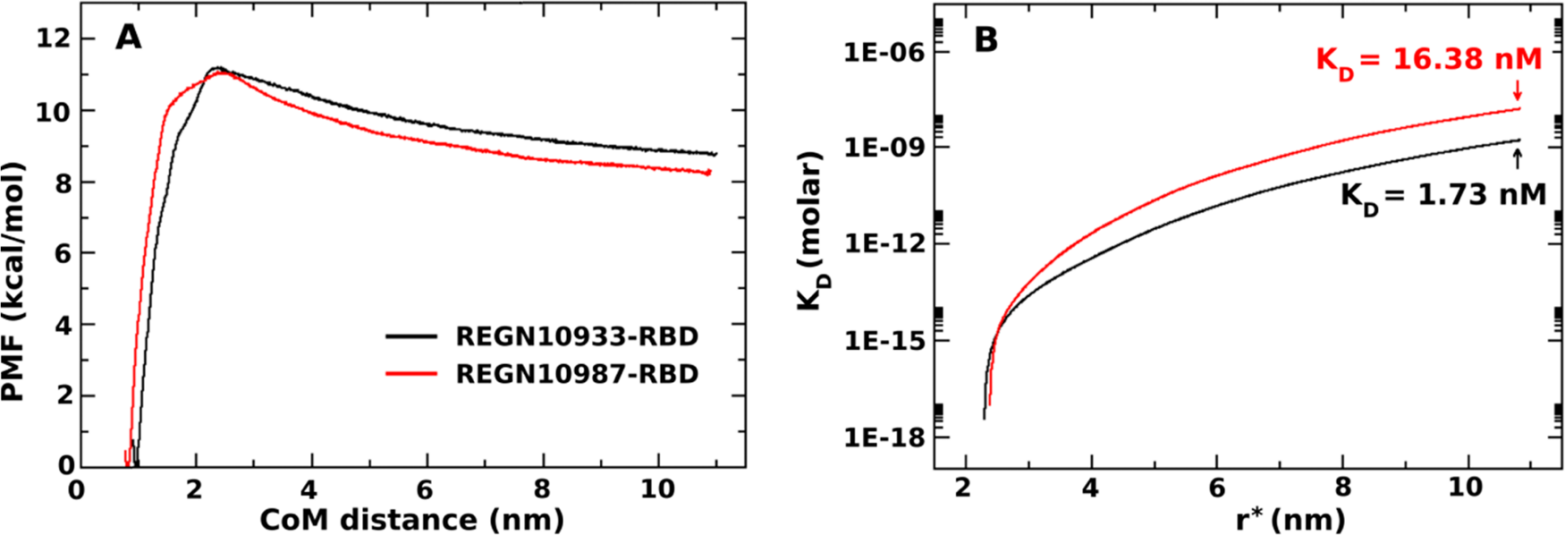
(Left) one-dimensional potential of mean force of REGN10933-RBD (black curve) and REGN10987-RBD (red curve). (Right) *K*_D_  curves as a function of r* corresponding to the change in the total free monomer concentration.

Figure 7A represents the 1D-PMF constructed from REX-US simulations. A barrier separating the bound and unbound regimes occurs at ≈2.3 nm for both complexes. Hence, we decided to choose *r*_b_ = 2.3 nm to numerically compute the probability *P*_b_ in eq 6. To evaluate the binding affinity of antibodies to the RBD domain, we calculate the dissociation constant *K*_D_ from eq 5. To solve eq 6, we need to determine a cutoff r* corresponding to a total volume limit to compute the probability of finding the system in the free monomer state and the free monomer concentration [A]. We select r* at around 11 nm as there is no longer an interaction between antibody and RBD beyond this threshold. *K*_D_ as a function of the distance r* tends to converge at large r* as expected (Figure 7B), and the approximately converged value was reported as *K*_D_ in our calculations. The results of *K*_D_ values for REGN10933 and REGN10987 binding to the RBD domain are listed in Table 1. As seen, the binding affinity of REGN10933 is stronger than that of RENG10987, and the difference is about 9–10 times. Our calculation is consistent with the experimental results of these monoclonal antibodies where the *K*_D_ were measured using surface plasmon resonance technology.^12^ From the experimental results, the *K*_D_ values of REGN10933 and REGN10987 are 3.37 and 45.2 nM, respectively, which means REGN10933 binds to RBD on the order of 13–14 times stronger than REGN10987.

### 3.4 PRODIGY Results

The free binding energy Δ*G*_bind_ calculated using PRODIGY is −10.7 ± 0.4 kcal/mol (*K*_D_ = 31 ± 8.96 nM) for REGN10933 and −10.2 ± 0.8 kcal/mol (*K*_D_ = 69 ± 25.33 nM) (Table 1) for REGN10987, implying that within the margin of error, this structure-based method cannot distinguish the binding affinity of REGN10933 from that of REGN10987. Therefore, PRODIGY is less accurate compared to our all-atom SMD and coarse-grained simulations, which show that, according to the experiment, REGN10933 binds to RBD more strongly than REGN10987. Applying PRODIGY to REGN10933+RENG10987-RBD, we obtained a binding free energy of −14.6 ± 1.0 kcal/mol (*K*_D_ = 0.056 ± 0.027 nM), which means that the cocktail can bind more tightly to the spike protein compared to its components. This result is consistent with the SMD result.

## 4 Conclusions

We studied the association of REGN10933 or REGN10987 or both REGN10933+REGN10987 with RBD of the SARS-COV-2 spike protein. The SMD results show that REGN10933 binds to RBD more strongly than REGN10987, which is consistent with the result calculated from coarse-grained REX-US. These computational results are in good agreement with the experimental results of Hansen et al.^12^ Moreover, SMD modeling and PRODIGY-based evaluation demonstrated that the REGN10933+REGN10987 cocktail tethers to RBD with higher affinity than either REGN10933 or REGN10987 alone, suggesting that this cocktail is more capable of preventing viral activity than its components.

The stabilities of REGN10933-RBD and REGN10933+REGN10987-RBD are mainly contributed by electrostatics interactions, while the stability of REGN10987-RBD is decided by vdW interactions. Lys417(A), Glu484(A), and Phe486(A) residues of the spike protein were found to play a crucial role in the binding affinity for the REGN10933 antibody, which may contribute to its neutralizing ability.

We show that REGN10933 and REGN10933+REGN10987 seem to have a similar activity for the Delta variant and WT. However, they are not effective against the Omicron variant, which is consistent with recent experiments.^20−22,45^

## Supporting Information Available

Hydrogen bonded and nonbonded contact networks of (A) REGN10933-RBD and (B) REGN10987-RBD complexes (Figure S1); structures obtained by molecular docking for ACE2 in combination with REGN10987, REGN10933, and RBD of WT and Delta (Figure S2); η values determined for stabilities of protein domains and interfaces, as well as for interactions between antibodies and RBD in CG simulations (Table S1); mutations in RBD of Delta and Omicron variants; the name of the lineage is also displayed; and total charge of RBD for WT, Delta, and Omicron as well as antibodies REGN10933 and REGN10987 (Table S3) (PDF)
