# Supplementary material for: Cocktail of REGN Antibodies Binds More Strongly to SARS-CoV-2 Than Its Components, but the Omicron Variant Reduces Its Neutralizing Ability
Source: J Phys Chem B. 2022 Apr 11;126(15):2812–23. doi: 10.1021/acs.jpcb.2c00708 (PMC9016775; doi:10.1021/acs.jpcb.2c00708)
Supplement: Supplementary file 1 — jp2c00708_si_001.pdf [file jp2c00708_si_001.pdf]

Supporting Information

Cocktail of REGN Antibodies Binds More Strongly to SARS-CoV-2 Than Its Components, But The Omicron Variant Reduces Its Neutralizing Ability

Hung Nguyen[1,+], Pham Dang Lan[2,3,+], Daniel A. Nissley[4], Edward P. O'Brien[5, 6, 7], and Mai Suan Li[1,*]

[1]Institute of Physics, Polish Academy of Sciences, al. Lotnikow 32/46, 02-668 Warsaw, Poland

[2]Life Science Lab, Institute for Computational Science and Technology, Quang Trung Software City, Tan Chanh Hiep Ward, District 12, 729110 Ho Chi Minh City, Vietnam

[3]Faculty of Physics and Engineering Physics, VNUHCM-University of Science, 227, Nguyen Van Cu Street, District 5, 749000 Ho Chi Minh City, Vietnam

[4]Department of Statistics, University of Oxford, Oxford Protein Bioinformatics Group, Oxford OX1 2JD, United Kingdom

[5]Department of Chemistry, Penn State University, University Park, Pennsylvania 16802, United States

[6]Bioinformatics and Genomics Graduate Program, The Huck Institutes of the Life Sciences, Penn State University, University Park, Pennsylvania 16802, United States

[7]Institute for Computational and Data Sciences, Penn State University, University Park, Pennsylvania16802 , United States

[+]These authors contributed equally

*Email: *masli@ifpan.edu.pl*

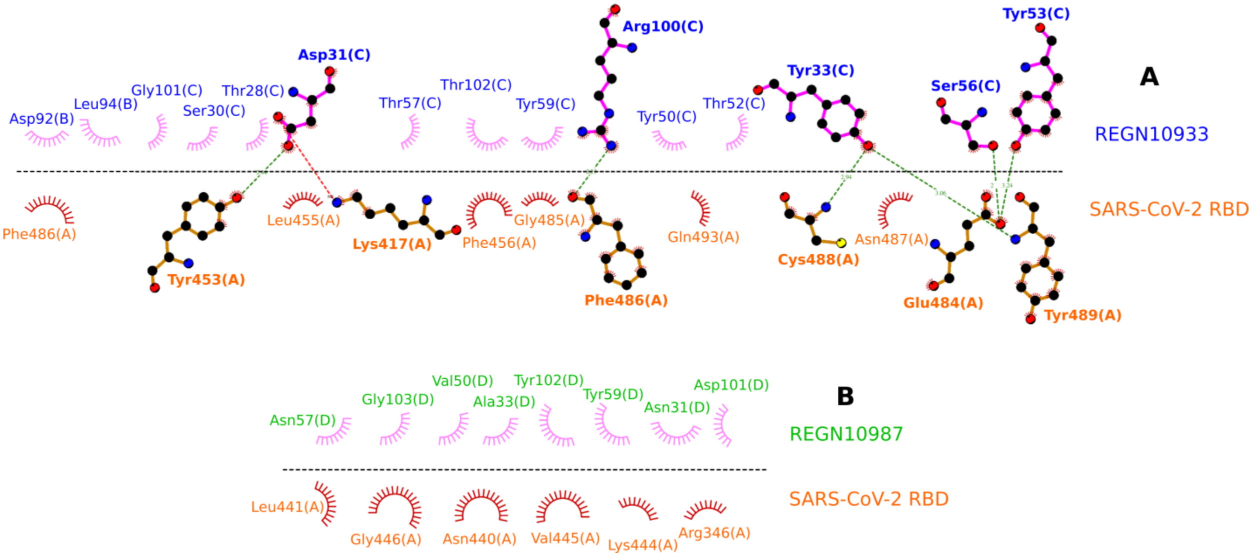

Figure S1: The hydrogen bond and non-bonded contact networks of (A) REGN10933-RBD and (B) REGN10987-RBD complexes.

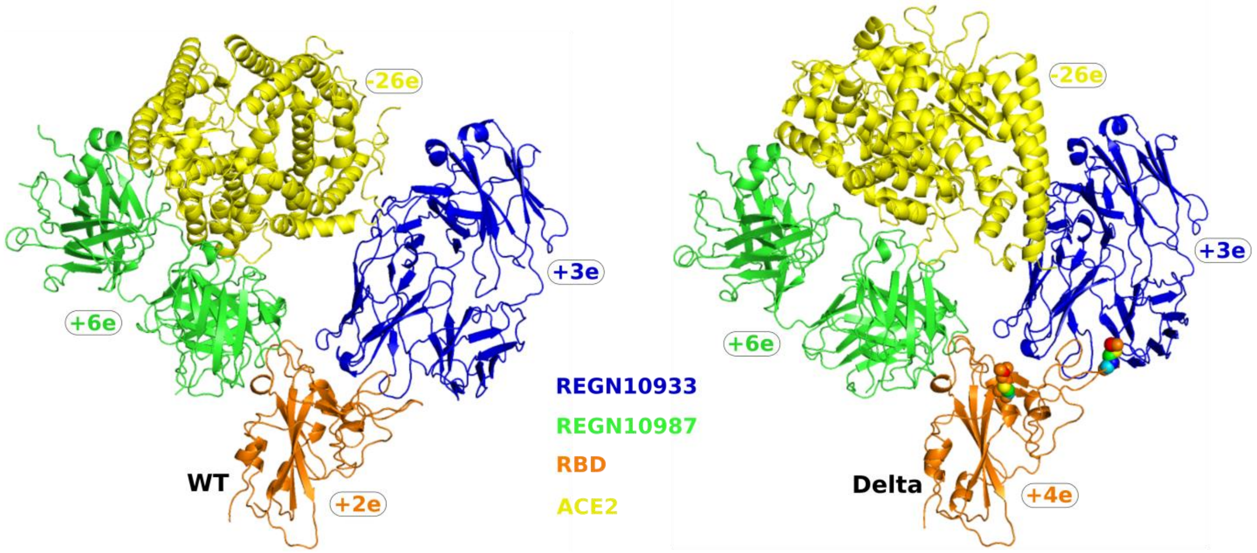

Figure S2. ACE2 (gold) in combination with REGN10987 (green), REGN10933 (blue) and WT RBD (left) and Delta RBD (right). Next to it is the charge of each molecule. The structures were obtained using HDOCK. The distance between the centers of mass of RBD and ACE2 is 2.37 nm and 2.19 nm for the WT and Delta cases, respectively.

Table S1: The η values determined for stability of protein domains and interfaces, as well as for interactions between antibodies and RBD in CG simulations.

	**REGN10933**	**REGN10987**	**RBD**	**REGN10933-RBD**	**REGN10987-RBD**
**η**	Chain L: 2.480<br>Chain H: 2.480<br>Interface: 2.124	Chain L: 2.480<br>Chain H: 2.480<br>Interface: 2.124	1.916	1.9	1.9

Table S2: Mutations in RBD of Delta and Omicron variants. The name of the lineage is also displayed. Blue refers to residues that have charge in RBD-WT, while red denotes residues that have charge after mutation.

**SARS-CoV-2 variants**	**Mutations in RBD**
India, **Delta** (B.1.617.2)	L452R, T478K

South Africa, **Omicron** (B.1.1.529)	G339D, S371L, S373P, S375F, K417N, N440K, G446S, S477N, T478K, E484A, Q493K, G496S, Q498R, N501Y, Y505H

Table S3: Total charge of RBD for WT, Delta and Omicron as well as antibodies REGN10933 and REGN10987.

	**Total charge (e)**
RBD-WT	+2
RBD-Delta	+4
RBD-Omicron	+5
REGN10933	+3
REGN10987	+6
